# Histological analysis of surgical lumbar intervertebral disc tissue provides evidence for an association between disc degeneration and increased body mass index

**DOI:** 10.1186/1756-0500-4-497

**Published:** 2011-11-16

**Authors:** Christoph Weiler, Mercedes Lopez-Ramos, H Michael Mayer, Andreas Korge, Christoph J Siepe, Karin Wuertz, Veronique Weiler, Norbert Boos, Andreas G Nerlich

**Affiliations:** 1 Institute of Pathology, Ludwig-Maximilians-University Munich, Germany; 2 Spine Center, Orthopaedic Clinic Munich-Harlaching, Germany; 3 Spine Research Group, Competence Center for Applied Biotechnology and Molecular Medicine, University Zurich, Switzerland; 4 AO Spine Research Network, Duebendorf, Switzerland; 5 Institute of Pathology, Academic Clinic Munich-Harlaching, Germany; 6 Institute of Pathology, Academic Clinic Munich-Bogenhausen, Germany

**Keywords:** disc degeneration, histologic degeneration score (HDS), body mass, ageing, surgical material

## Abstract

**Background:**

Although histopathological grading systems for disc degeneration are frequently used in research, they are not yet integrated into daily care routine pathology of surgical samples. Therefore, data on histopathological changes in surgically excised disc material and their correlation to clinical parameters such as age, gender or body mass index (BMI) is limited to date. The current study was designed to correlate major physico-clinical parameters from a population of orthopaedic spine center patients (gender, age and BMI) with a quantitative histologic degeneration score (HDS).

**Methods:**

Excised lumbar disc material from 854 patients (529 men/325 women/mean age 56 (15-96) yrs.) was graded based on a previously validated histologic degeneration score (HDS) in a cohort of surgical disc samples that had been obtained for the treatment of either disc herniation or discogenic back pain. Cases with obvious inflammation, tumor formation or congenital disc pathology were excluded. The degree of histological changes was correlated with sex, age and BMI.

**Results:**

The HDS (0-15 points) showed significantly higher values in the nucleus pulposus (NP) than in the annulus fibrosus (AF) (Mean: NP 11.45/AF 7.87), with a significantly higher frequency of histomorphological alterations in men in comparison to women. Furthermore, the HDS revealed a positive significant correlation between the BMI and the extent of histological changes. No statistical age relation of the degenerative lesions was seen.

**Conclusions:**

This study demonstrated that histological disc alterations in surgical specimens can be graded in a reliable manner based on a quantitative histologic degeneration score (HDS). Increased BMI was identified as a positive risk factor for the development of symptomatic, clinically significant disc degeneration.

## Background

Low back pain (LBP) is one of the major causes of pain and disability in the Western world, with a constantly rising life-time prevalence of approximately 60 to 85% [1,2], thus causing direct and indirect socioeconomic costs of up to $118.8 billions per year in the United States (US) alone [3]. Despite the high prevalence, LBP has still retained certain "enigmatic aspects" in terms of cause, diagnosis and treatment. Although several studies concerning the assessment of risk factors for LBP have been undertaken in the last couple of years, no distinct evidence has been provided [4]. In spite of the somehow inconsistent results of these investigations, there is growing evidence that age, gender, height, obesity, smoking, occupational exposure, heredity and psycho-social factors may constitute risk factors of LBP. Degeneration of the intervertebral disc (IVD), which is more common in patients with LBP than in asymptomatic individuals [5-7], is affected by multiple occupational backgrounds and genetic predispositions [5,6,8,9]. In the majority of cases, especially in the clinical setting, disc degeneration is classified using imaging techniques [10-17], while large-scale investigations on the histomorphological changes in the IVD, particularly in clinically well defined surgical material, are sparse. To our knowledge, the available histomorphological reports are predominantly based on post-mortem samples [18-26]. There seem to be two major reasons for this small number of investigations: (1) An accepted, reliable and feasible histological grading system to assess the morphological changes (in contrast to the broadly accepted MRI-classification systems) has not existed so far. (2) A possible lack of clinical interest in the excised disc material due to unfavorable cost effectiveness and missing therapeutic consequences [27-30].

Recently, Boos et al. have developed a reliable classification system [31] that allows a (semi-) quantitative assessment of histologic disc alterations in complete sagittal lumbar motion segments. This classification system has been established on a post-mortem cohort and validated on a small series of surgical samples. For the application of the histologic degeneration score (HDS) to the surgical material, the score had to be slightly modified. The criteria are described in Table I.

**Table 1:** Modified parameters collected for the histologic assessment of disc degeneration and scoring (Boos et al. 2002[31])

Criteria	*Grading*
*cell density (chondrocyte proliferation)*: multiple chondrocytes growing in small rounded groups or clusters sharply demarcated by a rim of territorial matrix	0 = no proliferation 1 = increased cell density 2 = connection of two chondrocytes 3 = small size clones (several chondrocytes grouped together, 3-7 cells) 4 = moderate size clones (8-15 cells) 5 = huge clones (> 15 cells)

*structural alterations (tears and clefts)*: concentric tears following the collagen fiber bundle orientation in the annulus fibrosus or radiating defects extending from the nucleus pulposus to the outer annulus lamellae parallel or oblique to the end-plate (clefts)	0 = absent 1 = rarely present 2 = present in intermediate amounts between 1 and 3 3 = abundantly present 4 = scar/tissue defects

*granular changes*: eosinophilic-staining amorphous granules within the fibrocartilage matrix	0 = absent 1 = rarely present 2 = present in intermediate amounts between 1 and 3 3 = abundantly present

*mucous degeneration*: cystic, oval or irregular areas with intense deposition of acid mucopolysaccharides (i.e. sulfated glycosaminoglycans) staining dark blue with Alc-PAS	0 = absent 1 = rarely present 2 = present in intermediate amounts between 1 and 3, 3 = abundantly present

Histologic Degeneration Score (HDS)	0-15 points

Although the histological examination of excised human tissue is mandatory (US) or highly recommended (Europe), there is nevertheless a growing informal consent in the surgical community to stop doing these analyses. In contrast, we believe that the histological examination of disc tissue allows a proper assessment of histo-degenerative changes and serves as a document for medicolegal purposes and quality control.

The aim of this clinicopathological study was to characterize the degree of disc degeneration in a large patient population in biopsies obtained from specialized spine centers using the modified histologic degeneration score (HDS) and to correlate findings with age, gender, and BMI.

A total of 854 disc samples referred to a pathology institute for routine histological assessment was analyzed in order to 1) Assess the reliability and practicability of a recently proposed histologic degeneration score (HDS) for the assessment of disc degeneration on excised disc material. 2) Investigate the clinical relevance of this grading in a risk factor analysis.

## Methods

### Study population and clinical data

The present clinicopathological study was conducted on a patient cohort that was treated in specialized orthopaedic spine centers covering the time period between 2003 and 2006. On the basis of available data, a total of 854 lumbar disc specimens was obtained during surgical procedures (i.e. discectomies, sequestrotomies, complete disc replacements etc.). The sex ratio was 529 (male): 325 (female).

In all cases, a HDS score (see below) could be determined, however, not all samples covered both nucleus pulposus (NP) and annulus fibrosus (AF) tissue. In 766 cases, part of the NP could be investigated, in 714 cases the AF was evaluable and in 626 cases, both tissue types were available for analysis.

In all cases, basic clinical data such as age and gender were available. A retrospective review of the patient information with regard to reliable data on preoperative body height and weight was successful in 249 cases (102 female/147 male). The main reason for this reduced number of data sets is either a lack of data in the records or retrospective measurements postoperatively.

On the basis of this data, the Body Mass Index (BMI (kg/m^2^)) (range 17,6-43,2) was calculated and the patients were grouped into 4 categories: I (underweight) < 20/II (Normal Weight) 20 < = 25/III (Overweight) 25 < = 30/IV (Obesity) > 30. A further subdivision in relation to female and male was unreasonable due to the resulting small numbers in each group.

The investigation was undertaken in accordance with the local ethic committee guidelines and was approved by the Ethical board of the Ludwig-Maximilians-University Munich.

### Tissue preparation

All surgical samples were immediately fixed in 4-6% buffered formaldehyde, pH 7.4 for approximately 12 - 16 hours. Cases with obvious calcification or residual bone material were gently decalcified in 0.1 M EDTA, pH 7.4 until complete decalcification.

The paraffin-embedded specimens were cut (2-4 μm) in slices, placed on silanized glass slides for routine staining (H&E, Masson-Goldner or Elastica-van Gieson's connective tissue stain, Alcian blue-PAS) and evaluated by light microscopy. A histomorphological distinction between annular and nuclear disc tissue was performed by use of light microscopic criteria particularly under polarized light, allowing the evaluation of the organization of the collagen network [31].

### Data evaluation

**Figure 1:**
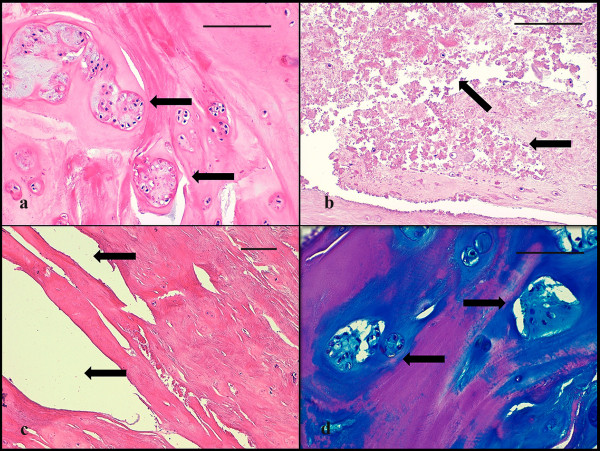
**Histomorphological signs for disc degeneration**. **a **increase of cell density (chondrocyte proliferation) with moderate clones of chondrocytes (arrows) in the NP of a 42 years old patient (score 4). **b **severe occurrence of granular changes (arrows) in the NP of a 67 years old patient (score 3). **c **structural alterations with tears and clefts (arrows) in the AF of a 54 years old patient (score 3). **d **severe increase in acid mucopolysaccharides (mucous degeneration) with dark blue staining areas around clones of chondrocytes (arrows) in the NP of a 77 years old patient. (a-c H&E stain; d Alcian blue-PAS stain/a-d scale bar 100 μm).

Patient samples were classified according to age and gender (854 cases). In a subset of these patients, we investigated the correlation of the HDS to age, gender, weight, height and BMI. All surgical specimen were classified according to the histologic degeneration score (HDS) [31] as described earlier. Briefly, the following parameters were used for grading (Table 1 and Figure 1): extent of cellularity (Figure 1a), structural changes of granular matrix degeneration (Figure 1b), the formation of clefts and tears (Figure 1c) and mucoid matrix changes (Figure 1d). Further parameters that had initially been evaluated in the autopsy series, such as necrosis, rim lesions etc., were omitted for the surgical samples since these parameters could not be evaluated with sufficient accuracy [31]. All data was obtained as a summary score that was recorded for each patient and each tissue type (nuclear vs. annular tissue).

Due to the variability of alterations inter-individually (and obviously different disc levels intra-individually), all investigated patients were grouped either according to their pre-operative BMI (if recorded, 4 groups) or according to their age (9 groups)

BMI: I (underweight) < 20/II (Normal Weight) 20 < = 25/III (Overweight) 25 < = 30/IV (Obesity) > 30

Age: I 10 < 20/II 20 < 30/III 30 < 40/IV 40 < 50/V 50 < 60/VI 60 < 70/VII 70 < 80/VIII 80 < 90/IX 90 < 100

### Statistical analysis

Differences between the investigated parameters were analyzed by Kruskal-Wallis one-way analysis of variance. The Kolmogorov-Smirnov test was used to compare the two patient samples in relation to age and gender. Correlations between the amount of histological changes with age, gender and BMI were explored using the Spearman rank test (statistical software SPSS 17.0). The level of significance was set to p < 0.05.

All gradings were assessed by two independent pathologists. The interrater reliability and intraobserver reliability of the histologic assessment of the variables was assessed on 100 respectively 50 randomly selected specimens by two of the authors (C.W. and A.G.N.) using kappa statistics.

## Results

### Sample Demographics

#### Total patient cohort

The descriptive statistic data for all patients are presented in Table 2. Gender comparison revealed that significantly more men (529) than woman (325) were involved (p < 0.0001), with a prevalence ratio for male to female of 1: 0.61. The patient age ranged between 15 and 96 years (mean 56 years).

**Table 2:** Patient sample data

Number	854
Male	529

Female	325

age range/mean [years]	15-96/56

Spine site lumbar	854

Spine level L1/L2	11

Spine level L2/L3	41

Spine level L3/L4	98

Spine level L4/L5	274

Spine level L5/S1	263

no information about the spine level	167

Samples with NP-HDS available	766

Samples with AF-HDS available	714

Samples with NP and AF-HDS available	626

Samples with additional BMI	249

#### Subset of patients with available BMI

Retrospective clinical data assessment revealed additional clinical information on weight and height for a subset of patients (249). The subset of patients (age range 21 to 96 years; mean 54 years) with available BMI showed a comparable age and gender distribution. Significantly more men (147) than woman (102) were involved (p < 0.0001), with a prevalence ratio of 1: 0.69 (almost identical to the whole study group).

The Kolmogorov-Smirnov test revealed no statistically significant differences (p > 0.05) between the two patients groups in relation to age and gender.

#### Patient distribution over sampling time (2003-2006)

**Figure 2:**
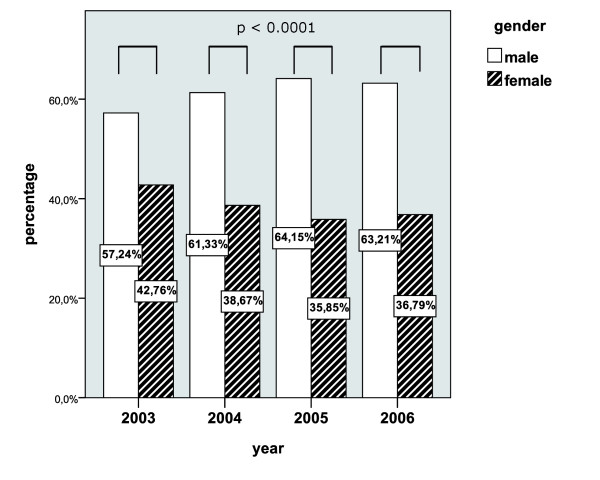
**Distribution of both sexes in relation to the investigated years (relative numbers per year in percentage)**.

Over the entire sampling time (2003-2006), the comparison of both sexes in terms of relative occurrence revealed an equal fraction with a relative occurrence for women between 35.85 to 42.76% and for men between 57.24 to 64.15%. These small differences in relative occurrence over the different years showed no statistical significance (see Figure 2).

#### Age distribution

**Figure 3:**
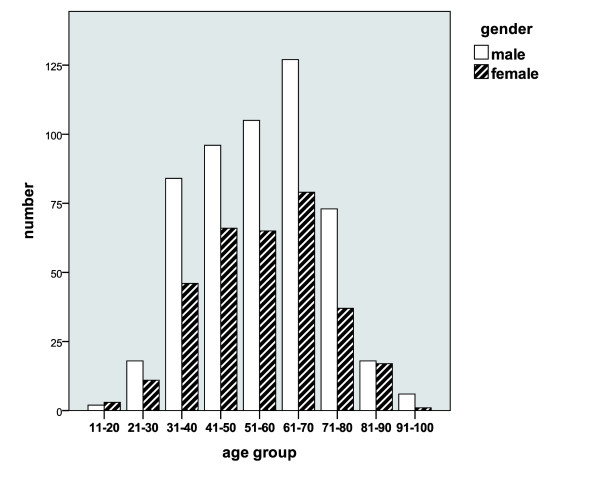
**Age distribution of the patient population in absolute numbers**.

With regard to the patient age allocation, an almost normal curve of distribution is seen with an accumulation in the age period 30 to 80 years and a peak incidence in age decade 61 to 70 years for both genders (see Figure 3).

### Histomorphological analysis

The application of the histologic degeneration score (HDS) in daily care routine practice was easy and feasible and did not significantly prolong the analysis time. The assessment of the parameters cell proliferation, granular changes and mucoid changes was very reliable and straight forward even in small tissue samples. Only in few, very fragmented samples, structural alterations (tears/clefts) were difficult to assess and may thus lead to certain inaccuracy.

#### Histologic Degeneration Score (HDS)

The histo-morphological appearance of the disc material was almost identical to that described in previous studies on surgical material [31,32]. It was possible to reliably apply the HDS in all cases (n = 854) on the tissue fragments obtained during surgical interventions. A distinction between annular (AF) and nuclear (NP) disc material was determined on the basis of histomorphology, particularly by use of birefringence of the collagen network [31].

**Figure 4:**
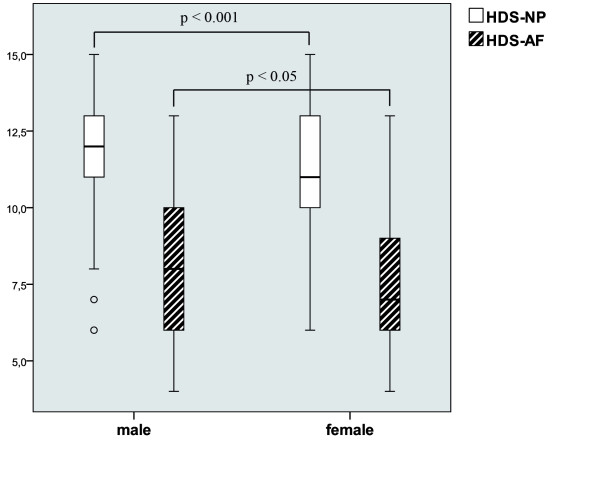
**Histologic Degeneration Score (HDS) of the annular (AF) and nuclear (NP) region in relation to gender (Box-Plot)**.

In general, the histological changes were accentuated in NP tissue with a significantly higher HDS in the NP (mean 11.45) than in the AF (mean 7.87) (p < 0.00001). Comparison of both sexes in relation to the analyzed HDS (see Figure 4) showed significant higher levels in males with a mean HDS in the NP of 11.63 and in the AF with 8.0 compared to females with a mean HDS in the NP of 11.16 and in the AF with 7.67. These differences were statistical significant for the NP (p = 0.0005) and AF (p = 0.029).

#### Reliability of the Histologic Assessment

Calculation of the interrater reliability for the assessment of the NP-HDS and AF-HDS in 100 randomly selected cases in general showed excellent rater agreement (Kappa statistics: NP-HDS = 0.836; AF-HDS = 0.846).

The intraobserver reliability test showed excellent intra-rater agreement (Kappa statistics: C.W. = 0.849; A.G.N. = 0.868).

#### Histologic Degeneration Score and Body Mass Index

**Figure 5:**
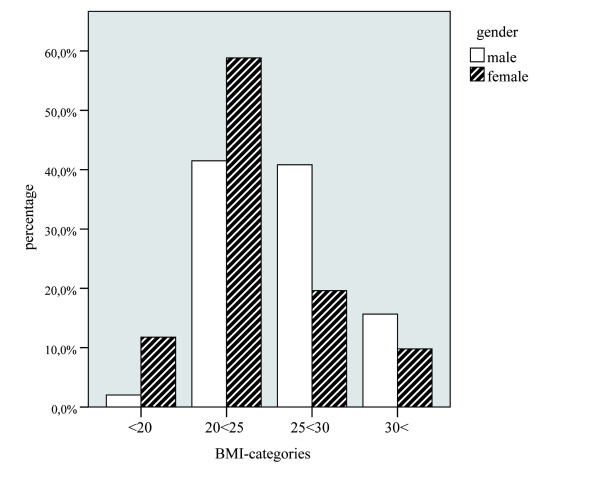
**Distribution of male and female patients in relation to the Body mass index (BMI) (relative numbers in percentage)**.

**Figure 6:**
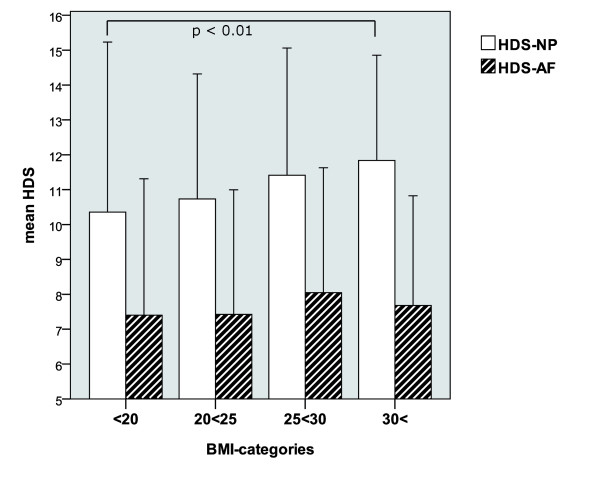
**Interrelation of the annular (AF) and nuclear (NP) HDS with the BMI (mean +/- 2 Stdev)**.

In 249 patients, weight and height were available for the generation of the pre-operative body mass index (BMI). After categorization of the BMI values (range 17.6 - 43.2) in 4 groups, we found an accumulation of patients, in particular with regard to male sex (see Figure 5) and higher BMI levels (see Figure 6). The relation between the amount of the histologic degeneration score (higher HDS) and the BMI revealed statistical significance (p = 0.001) with higher HDS values in the NP for obese patients.

#### Histologic Degeneration Score and age distribution

**Figure 7:**
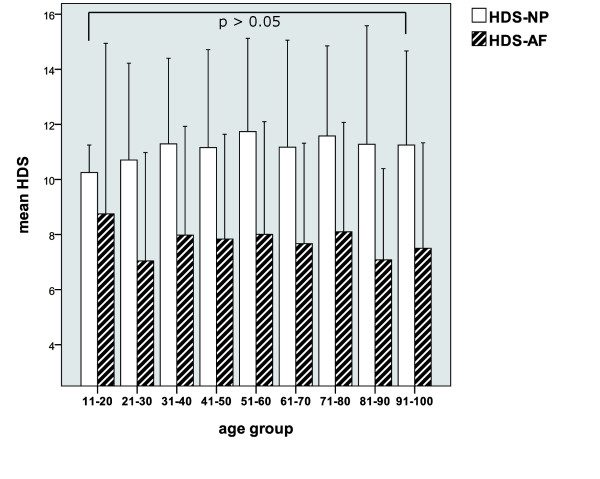
**Correlation of the annular (AF) and nuclear (NP) HDS with patient age groups (mean +/- 2 Stdev)**.

According to their age, all patients were categorized into 9 different age groups (range 15-96 years). The extent of histologic disc changes did not show a significant age related pattern (see Figure 7).

#### Statistical correlation

The results of the correlation of the HDS with the investigated clinical parameter are presented in Table 3. As described, we found a highly significant correlation between the BMI and the extent of histomorphological changes in the NP (p = 0.001) and to a lesser extent in the AF (p = 0.010).

**Table 3:** Statistical correlation (Spearman-Rho, two-tailed)

	Statistic	HDS-NP	HDS-AF	age	weight	height	BMI
HDS-NP	coefficient of correlation	1.000	.328**	.051	.233**	.154*	.222**

	level of significance	.	.000	.155	.000	.018	.001

HDS-AF	coefficient of correlation	.328**	1.000	-.002	.124	-.027	.177**

	level of significance	.000	.	.949	.071	.694	.010

age	coefficient of correlation	.051	-.002	1.000	-.072	-.320**	.172**

	level of significance	.155	.949	.	.254	.000	.006

weight	coefficient of correlation	.233**	.124	-.072	1.000	.662**	.820**

	level of significance	.000	.071	.254	.	.000	.000

height	coefficient of correlation	.154*	-.027	-.320**	.662**	1.000	.157*

	level of significance	.018	.694	.000	.000	.	.013

BMI	coefficient of correlation	.222**	.177**	.172**	.820**	.157*	1.000

	level of significance	.001	.010	.006	.000	.013	.

**. correlation on the p = 0.01 level of significance

*. correlation on the p = 0.05 level of significance

## Discussion

Although there have been many experimental studies on disc material - mostly post-mortem tissue samples in animal experimental models - and some investigations on human post-mortem cohorts, little information on the quantitative amount of histomorphological changes in surgical material is available. Several histological studies [33-36] have shown the occurrence of granulation tissue with an abundant neovascularisation as a hallmark of prolapsed and protruded disc material. Weidner et al. [36] stated that the occurrence of edge neovascularisation is a reliable histological clue that intervertebral disc prolapse has occurred. However, this has been refuted by several authors as no significant differences were seen between sequestered and non-sequestered material with respect to neovascularisation in these studies [24,37]. Other studies have focussed on the composition of the herniated material, which has been the subject of several histological studies [19-21,23,25,38]. The results of these studies are conflicting with different statements about the composition of herniated material (annular, nuclear and endplate). These contradictory observations may partly result from different histological criteria used for the distinction between nuclear and annular tissue. Few of these studies [19,20,25] have correlated the histological composition of the herniated disc material with clinical outcome. While some authors did not find any correlation between histological composition and clinical outcome [19,25], others found an association with increased pain intensity and clinical outcome [20]. Furthemore, there is an ongoing widely unresolved dispute [39] concerning the interplay of the different intervertebral disc compartments in the process of disc degeneration and sciatica. Especially the discussion concerning the chronology of observed disc alterations, such as weakening of the annulus fibrosus with secondary degeneration of the nucleus pulposus or synchronous degeneration of both compartments or vice versa is still under examination [26,40,41].

The scope of our investigation was the assessment of histological alterations of disc tissue in routine pathology on the basis of a specialized histologic degeneration score (HDS) [31,42]. Although we examined surgical - mostly fragmented - disc material, we were able to establish a HDS in all available cases. In accordance with previous investigations [31,32,42], we found similar results concerning the occurrence of histological changes in the different disc compartments with more pronounced changes in the nucleus pulposus. We are aware of the fact that the allocation of the scoring system (initially developed for whole intervertebral discs) to surgical material might lead to some imprecision. Some of the assessed factors are weighted to different degrees, for instance granular changes could be underestimated due to "wash out" effects and on the other hand, tears and clefts could be overestimated due to the mostly fragmented character of the specimens. However, both the present study as well as previous evaluations [31,42] for example in a post-mortem analysis and a small surgical specimen study [32] strongly supports the notion that the HDS can also be applied to surgical tissue in a reliable manner. The only slight modification that had to be taken into account covered few criteria that cannot be evaluated in a safe and reliable manner in the fragmented surgical tissue, such as rim lesions or necrosis.

This is, to our knowledge, the first study which demonstrates a significant correlation between the extent of the histologic degeneration score (HDS) and the pre-operative BMI. Previous investigations [43-60], which were mainly based on radiological criteria, showed heterogeneous results although the majority [44-51,54,58,61] favours obesity or increased BMI as a risk factor. Interestingly, despite our extensive literature review, we could not find any study that takes the amount of histopathological changes in the surgical excised material into account, possibly because of a missing classification system.

Although genetic and epidemiologic studies in the past provide some evidence for a hereditary background [5,8,9,13,62-65], several occupational or lifestyle related potential risk factors have been identified [46,66-69]. Even though the assessment of this epidemiologic data is difficult and the results of these surveys are partially controversial, there is a strong belief that obesity plays an important role [4,44,47,51,54,60,61,66,70] among the potential risk factors. From a biomechanical point of view, mechanical overstraining is sufficient to explain the possible association to LPB or disc degeneration. We fully agree with the point that biomechanics plays a role in disc degeneration, but from our experience [32,42,71], there might be another - perhaps more important - influence in terms of a dysregulation of the metabolic and immune system [72]. For example obesity, insulin resistance and type 2 diabetes are closely associated with chronic "inflammation" and characterized by an abnormal cytokine production and activation of a network of inflammatory signalling pathways, e.g. leading to an overexpression of TNF-α in the tissue of obese humans [73,74]. Interestingly, there is substantial evidence [32,42,71,75-79] that inflammatory cytokines play a significant role in the process of accelerated disc degeneration. One might speculate that obesity could possibly influence the process of disc degeneration through two - perhaps synergistic - pathways. Disc degeneration is a multifactorial process involving both environmental and genetic factors, synergistic effects have already been shown in a gene-environment interaction by Solovieva et al. [60].

Why should we classify histological changes in excised disc material? There is a substantial bulk of literature [27,29,30,80,81] suggesting that routine histopathological examination of disc specimen is not justified for reasons of cost effectiveness. However, taking the results of the present study into account, we provide clear evidence that the determination of the histologic degeneration score (e.g. by the HDS) in the clinical setting is reliable and feasible with regard to its requirements (personnel, equipment), work load and costs and thus presents important data on the constitution of the sample. This morphological data might be of importance because it possibly allows an inference about the status of disc degeneration in the remaining intervertebral discs. Secondly, the evaluation of histo-degenerative changes in the excised disc material serves as a document for medicolegal purposes and quality control.

Finally, future therapeutic consideration, for example by selective inhibition of inflammatory cytokines or cell based transplantation therapies, will require a morphologic rationale for those therapies. In these cases, detailed information about the current status of intervertebral disc tissue might be mandatory to choose the right therapeutic options.

## Conclusions

This study describes the histomorphological features in excised disc material from a large orthopaedic spine center patient population (854 patients) that have been histopathologically evaluated by two histopathology units specialized in the evaluation of disc pathology. With this study, we provide evidence that a previously (on autopsy samples) established and validated histologic degeneration score (HDS) can be applied to surgically obtained disc material and can reliably be integrated in daily care routine pathological evaluation. Furthermore, in agreement with our previous findings, we were able to show that characteristic histological degenerative disc changes were more pronounced in the NP than in the AF. Statistical analysis demonstrated a gender imbalance with a significantly higher HDS in males. For a subset of patients with available BMI, we could detect a positive correlation between BMI and HDS, substantiating an accelerated course of disc degeneration in obese individuals.

## Competing interests

The authors declare that they have no competing interests.

## Authors' contributions

CW and AGN were the main composer of the manuscript. MLP and VW were involved in data analysis. KW and NB participated in the study design and editing of the manuscript. HMM, AK and CJS were involved in conception of the study and performed surgery. All authors read and approved the final manuscript.
